# Incidence and Outbreak of Healthcare-Onset Healthcare-Associated *Clostridioides difficile* Infections Among Intensive Care Patients in a Large Teaching Hospital in China

**DOI:** 10.3389/fmicb.2018.00566

**Published:** 2018-03-27

**Authors:** Chunhui Li, Yuan Li, Yang Huai, Sidi Liu, Xiujuan Meng, Juping Duan, John D. Klena, Jeanette J. Rainey, Anhua Wu, Carol Y. Rao

**Affiliations:** ^1^Infection Control Center, Xiangya Hospital Central South University, Changsha, China; ^2^International Emerging Infections Program, Division of Global Health Protection, Center for Global Health, U.S. Centers for Disease Control and Prevention, Beijing, China; ^3^Division of Global Health Protection, Center for Global Health, U.S. Centers for Disease Control and Prevention, Atlanta, GA, United States

**Keywords:** *Clostridioides difficile* infection (CDI), incidence, outbreak, antibiotic associated diarrhea (AAD), global health security

## Abstract

**Background:** *Clostridioides difficile* infection (CDI) is an important cause of morbidity and mortality among hospitalized patients. In China, however, hospital staff do not routinely test for CDI, leading to under-diagnosis and poor patient outcomes. Locally generated CDI data can help assess the magnitude of the problem and strengthen approaches for CDI prevention and control.

**Methods:** We prospectively monitored hospital-onset hospital-associated (HOHA) CDI in four intensive care units (ICUs) from June 2013 to September 2014 in a large teaching hospital in China. We collected clinical information from all ICU patients with ≥ 3 episodes of diarrhea occurring within a 24-h period at least 48 h following admission (suspect case definition). Stool specimens were collected from all suspect cases of CDI and cultured for *C. difficile*. Polymerase chain reaction (PCR) was used to detect toxin genes from positive isolates; multi-locus sequence typing (MLST) was used for typing and identifying novel strains. We estimated the incidence rate as the number of HOHA CDI cases per 10,000 patient days; 95% confidence intervals were generated to assess rate differences between the four ICUs.

**Results:** A total of 593 hospital-onset diarrhea patients met the suspect case definition during the study period. Of these, 47 patients (8%) were positive for *C. difficile* and toxin genes. The HOHA-CDI incidence rate was 14.1 cases per 10,000 patient days (95% CI: 10.5–18.6). Six patients with HOHA CDI died. ST54 (*n* = 14, 20%) was the most common type of HOHA-CDI strain circulating in the hospital during the study period and was linked to a temporal cluster (outbreak) involving two (NICU and GICU) of the four ICUs.

**Conclusion:** HOHA-CDI occurs among ICU patients at this teaching hospital, supporting the importance of routine testing for CDI. Information on strain distribution can help detect CDI outbreaks. Detection of ST54 strain in a temporal cluster suggests possible gaps in infection control practices that should be investigated and addressed as needed.

## Introduction

*Clostridioides difficile* infection (CDI) is an important cause of hospital-associated infections (HAI) worldwide. Clinically, CDI can range from mild diarrhea to severe pseudomembranous colitis (PMC) (Yassin et al., 2001). Epidemics of CDI have occurred in North America and Europe over recent decades, and the epidemiology of CDI in these regions is generally well documented. These epidemics have been largely due to the hypervirulent *C. difficile* RT027/ST1, a strain frequently associated with increased mortality (Garey et al., 2008; Jamal et al., 2010; Abou Chakra et al., 2015). Common risk factors for CDI are well known and include hypervirulent strains, age, underlying conditions, and use of antibiotics, as well as immune suppression and exposure to CDI (Garey et al., 2008; Jamal et al., 2010; Lai et al., 2014; Abou Chakra et al., 2015).

In China, a country with more than 5,000 secondary and tertiary hospitals, CDI is currently not notifiable through national, provincial, or hospital-based surveillance systems (Zhang et al., 2016). Except for a few specific studies, information is scarce about the burden and strain distribution of CDI in the country. Two *C. difficile* 027 isolates were identified in Beijing from two patients coming for outpatient visits in 2012 and 2013 (Cheng et al., 2016). Physicians tend to rely on clinical assessments for identifying CDI cases, and laboratory confirmation for *C. difficile* is not routinely performed in most hospitals. Challenges in isolating CDI as well as the costs associated with identifying toxigenic strains create additional barriers to diagnostic testing. We suspect this lack of laboratory confirmation leads to under-diagnosis and poor patient outcomes, particularly among vulnerable patients in intensive care units.

To address this gap, we conducted a prospective surveillance project for CDI in intensive care units (ICUs) at Xiangya Hospital at Central South University, located in the city of Changsha in Hunan Province in central China. We anticipate that these locally generated CDI data will help to better assess the magnitude of the problem and strengthen approaches for CDI prevention and control as well as highlight the importance of laboratory-based surveillance.

## Materials and Methods

### Study Location

Xiangya Hospital is a 3,500-bed urban tertiary teaching hospital in Changsha, Hunan Province, China and admits approximately 90,000 patients per annum. The hospital has four non-neonatal ICUs, including the General ICU (GICU, 35 beds), Neurosurgery ICU (NSICU, 20 beds), Neurology ICU (NICU, 16 beds), and Respiratory ICU (RICU, 10 beds). We prospectively monitored patients in the four non-neonatal ICUs from June 2013 to September 2014 to identify cases of hospital-onset diarrhea.

### HOHA-CDI Case Definition

Staff in each of the four ICUs prospectively monitored all patients admitted for hospital-onset hospital-associated (HOHA) diarrhea, that is, diarrhea occurring ≥48 h after hospital admission and prior to discharge (McDonald et al., 2007). We considered hospitalized patients with ≥ three diarrhea episodes within 24 h as suspected HOHA-CDI cases and eligible for study participation (Cohen et al., 2010). Stool specimens were collected from these patients and tested for *C. difficile.* We defined suspected HOHA-CDI patients with a stool test positive for the presence of toxigenic *C. difficile* or colonoscopic or histopathologic findings demonstrating PMC as confirmed HOHA-CDI cases (Cohen et al., 2010).

Patients with suspected HOHA diarrhea and stool samples yielding positive results for toxin-producing *C. difficile* organisms by culture that were also PCR confirmed as *tcdB*-positive were defined as having HOHA-CDI. We excluded patients less than 18 years of age since intestinal tract colonization with *C. difficile* can be common among children. The confirmed case definition of HOHA-CDI is consistent with internationally recognized recommendations (McDonald et al., 2007). For the purpose of this study, we defined HOHA-CDI outbreaks as the detection of three or more cases in any of the ICUs with the same ST type or ribotype of HOHA-CDI within a 7-day period or five cases (same type) within a 4-week period.

### Laboratory Diagnostic Approaches

We collected stool specimens from patients once the case definition for suspected CDI was met. Specimens were cultured under anaerobic conditions (ANAEROGEN COMPACT AN0020C, OXOID, Basingstoke, United Kingdom) using *C. difficile* CDMN Agar (CM0601+SR0173, OXOID Basingstoke, United Kingdom). Colonies with a horse dung odor were subjected to Gram stain. Gram-positive bacilli with sub-terminal spores, and those that yielded positive results according to a commercially available latex agglutination test (DR1107A, OXOID, Basingstoke, United Kingdom) and PRO DISK (R211357, Remel, Lenexa, KS, United States) were identified as *C. difficile*. We confirmed identification using internationally established standard testing procedures, including 16S rRNA sequence analysis (Persson et al., 2008).

We prepared genomic DNA from *C. difficile* cultured on blood agar (BioMerieux, Shanghai, China) after 48 h at 37°C in anaerobic conditions. High molecular weight DNA was extracted using a High Pure PCR Template Preparation Kit (QIAamp DNA Mini Kit, QIAGEN, Valencia, CA, United States) according to the manufacturer’s instructions. We detected the toxin genes *tcdA, tcdB, cdtA*, and *cdtB* by PCR according to prior recommendations (Lemee et al., 2004; van den Berg et al., 2004; Pituch et al., 2005). Multi-locus sequence typing (MLST) was performed and analyzed for the toxigenic and non-toxigenic *C. difficile* strains using a previously established method (Griffiths et al., 2010). We linked specimen collection dates and test results to the hospital case-patient record data by the unique study ID.

### Data Collection

The hospital information system (HIS) of Xiangya Hospital is a comprehensive information management system containing patients’ demographic information as well as clinical and treatment details for each hospital stay. We used a standard form to abstract the following information from the HIS for each suspect CDI case-patient enrolled in the study: demographic data (age, gender, place of residence), dates of hospital and ICU admission, primary diagnosis, underlying conditions, invasive procedures and devices used, antibiotic prescriptions, laboratory tests, and health outcomes, as well as dates of ICU and hospital discharge. Record information was abstracted according to the case-patient’s hospital number and each patient was assigned a unique study ID. Data were entered into an EpiData (3.0 Denmark) database.

### Data Analysis

We linked epidemiological, clinical, and laboratory data by study ID, verified the data, and then imported the data into SAS 9.3 (Cary, NC, United States) for analysis. Patient demographic characteristics, underlying conditions and antibiotic usage prior to and during hospitalization were described. We calculated the incidence rate of HOHA-CDI as the number of confirmed HOHA-CDI case-patients divided by the total number ICU patient-days in the four ICUs during the study period. We generated 95% confidence intervals for the overall incidence rate as well as for the rate in each of the four ICUs. Fisher’s exact tests were performed to compare HOHA-CDI and non-CDI diarrhea patient characteristics. We used an alpha level of 0.05 to assess statistical significance. Characteristics of CDI-toxigenic strains isolated from patients, including specific molecular genetic traits, as well as the temporal distribution of these strains were described.

### Ethical Review

The Ethics Committee of the Xiangya Hospital of Central South University and the United States Centers for Disease Control and Prevention IRB authorization agreement approved the study protocol (CGH #2014-047). Since the study involved the collection and testing of stool specimens for CDI, only verbal consent from the patient (or a family member) was required for enrollment. We communicated laboratory results to patients’ attending physicians for care and treatment per hospital CDI guidelines (Cohen et al., 2010).

## Results

**FIGURE 1:**
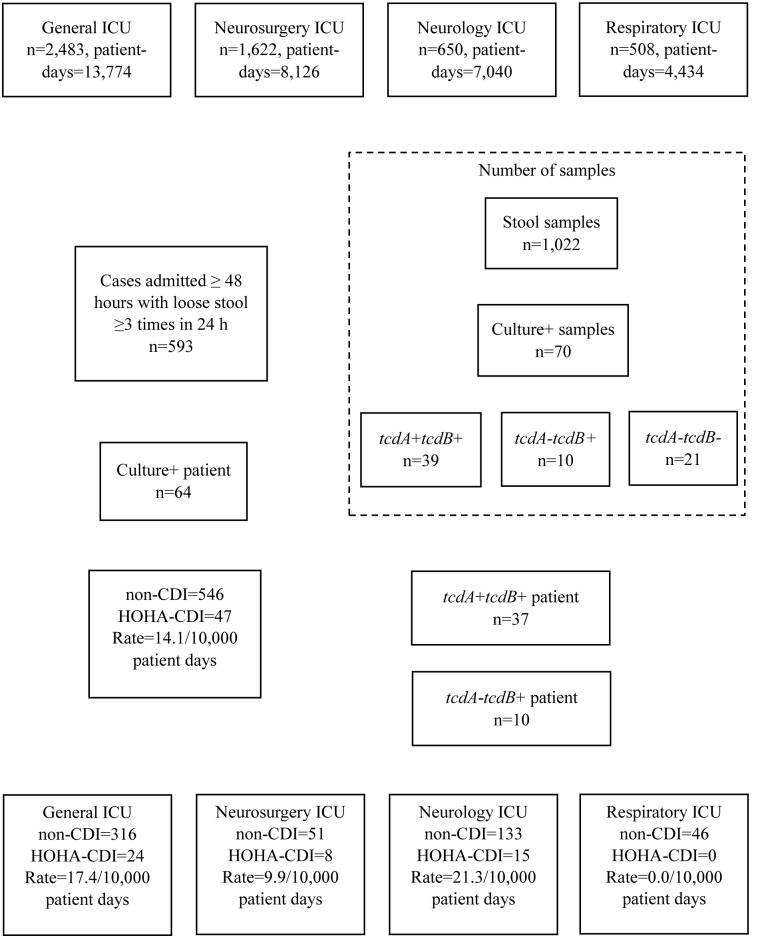
Identification and classification of suspected cases of HOHA-CDI among Intensive Care Unit patients, Xiangya Hospital, June, 2013 – September, 2014.

A total of 593 (11.3%) of the 5,263 patients hospitalized in the four ICUs met the suspected CDI case definition and were enrolled in the study (**Figure 1**). We collected 1,022 stool specimens from 593 suspected case-patients, of whom 47 were confirmed with HOHA-CDI. One additional suspected case-patient was determined to have community-onset CDI, and therefore, was not included in the case series. The majority of confirmed HOHA-CDI cases were between 41 and 64 years of age (*n* = 26, 55.3%) and male (*n* = 34, 72.3%) (**Table 1**).

**Table 1:** Demographic and clinical characteristics of suspected case-patients by CDI status^∗^, Xiangya Hospital, June 2013 – September 2014.

Demographic and clinical characteristics	HOHA-CDI (*n* = 47)		No HOHA-CDI (*n* = 546)		Fisher’s exact test
	*n*	%	*N*	%	*P*-value^∗∗^
Sex					
Female	13	27.7	174	31.9	0.6257
Male	34	72.3	372	68.1	
Age group					
18–40	6	12.8	96	17.6	
41–65	27	57.4	298	54.6	0.7021
>65	14	29.8	152	27.8	
Intensive care unit (ICU)					
General	25	53.2	316	57.9	
Neurosurgery	6	12.8	51	9.3	0.0993
Neurology	16	34.0	133	24.4	
Respiratory	0	0	46	8.4	
Antibiotic use during hospitalization					
Yes	47	100.0	520	95.2	0.2535
No	0	0.0	26	4.8	
Proton pump inhibitor (PPI) use					
Yes	45	95.7	473	86.6	0.1049
No	2	4.3	73	13.4	
Duration of hospitalization (in ICU)					
<2 weeks	10	21.3	116	21.3	0.9096
2 weeks–2 months	34	72.3	403	73.8	
>2 months	3	6.4	27	4.9	
Died during hospitalization					–
Yes	6	12.8	NA	NA	
No	41	87.2	NA	NA	
Underlying conditions^§^					–
Diabetes	8	17.0	80	14.7	
Malignancy	5	10.6	39	7.1	
Hypertension	15	31.9	209	38.3	
Hematopathy	2	4.26	6	1.1	
Respiratory failure	7	14.9	52	9.5	
HIV	0	0	0	0	
Renal insufficiency	5	10.6	44	8.1	
Coma	17	36.2	153	28.0	
Cardiac insufficiency	2	4.3	61	11.2	
Tuberculosis	1	2.1	12	2.2	
Treatments prior to hospitalization^§^					–
Antibiotic use	39	83..0	144	26.4	
Immunosuppressant use	0	0	11	2.0	
Glucocorticoid use	10	21.3	22	4.0	

*^∗^Suspected cases: All patients admitted to the four ICUs were monitored prospectively for hospital-onset hospital-associated (HOHA) diarrhea, that is, diarrhea occurring ≥48 h after hospital admission and without discharge. Non-HOHA-CDI patients met the HOHA-CDI suspect case definition but tested negative for toxin-producing CDI.*

*^∗∗^*P*-values were generated from Fisher’s Exact Test; an alpha level of 0.05 used to assess statistical significance.*

*^§^Patients could have more than one underlying condition as well as treatments prior to hospitalization; we did not perform statistical tests on these patient characteristics.*

All confirmed HOHA-CDI case-patients had one or more underlying conditions at the time of hospitalization. The majority received antibiotics within 30 days of hospitalization (*n* = 39, 83.0%). All case-patients received antibiotics during hospitalization; almost half of the patients (*n* = 23, 48.9%) had received carbapenems. Of the 47 confirmed CDI case-patients, 45 (9.7%) were also treated with proton pump inhibitors (PPIs). The mean length of hospital stay was 29.1 days (range: 2 to 225). During the 15-month study, six (12.8%) of the HOHA-CDI patients died. Three patients died as a result of CDI (severe diarrhea and toxic megacolon), and the other three died from their initial underlying disease (i.e., central nervous system infection, respiratory failure and chronic obstructive pulmonary disease). Patient characteristics of HOHA-CDI and non-CDI diarrhea cases had statistically similar patient characteristics (**Table 1**).

### Incidence Rates

**FIGURE 2:**
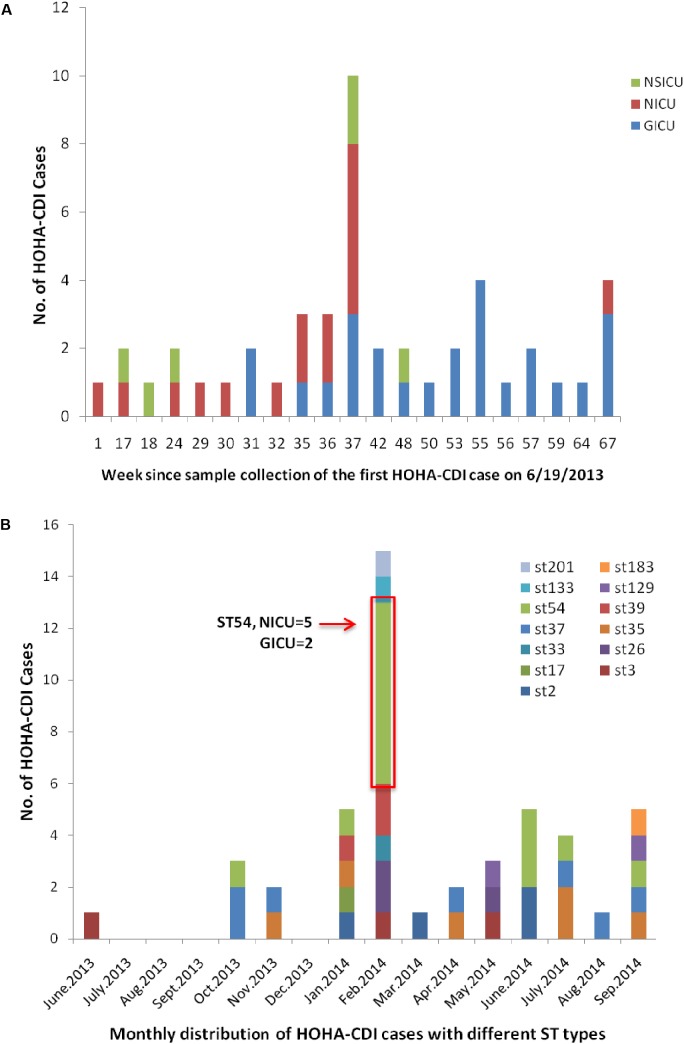
Temporal distribution and outbreak of HOHA-CDI. **(A)** Weekly distribution of all HOHA-CDI cases among Intensive Care Unit patients. **(B)** Monthly distribution of HOHA-CDI cases among Intensive Care Unit patients. ST54 case cluster occurring in the NICU (*n* = 5) and GICU (*n* = 2) in February 2014 attributed CDI outbreak.

The overall incidence of HOHA-CDI was 14.1 per 10,000 patient days (95% CI: 10.5–18.6) during the 15-month study period (**Figure 2A**). The incidence rate in the NICU and GICU were 21.3 per 10,000 patient days (95% CI: 12.4–34.4) and 17.4 (95% CI: 11.4–25.5), respectively, both higher than in the NSICU (9.9 cases per 10,000 patient days, 95% CI: 4.6–18.7), and in the RICU (0 cases per 10,000 patient days).

### Molecular Characteristics

Of the 1,022 specimens, 70 were culture-positive for *C. difficile*; of these, 49 were positive for either *tcdA* or *tcdB* gene. Twenty-one were negative for both *tcdA* and *tcdB.* A total of 17 different sequence types (STs) were observed by analyzing all isolates including toxigenic and non-toxigenic *C. difficile* strains by MLST. ST54 (*n* = 14, 20%) was the most common MLST type, followed by ST39 (*n* = 10), ST26 (9), ST37 (7), ST35 (7), ST3 (5), ST2 (4), ST48 (2), ST17 (2), ST15 (2), ST129 (2), ST201 (1), ST33 (1), ST133 (1), ST183 (1), ST274 (1), and ST296 (1). Neither ST1 (BI/NAP1/027) nor ST11 (ribotype 078) was detected during the study period. All ST54, ST35, and ST2 strains were toxigenic and belonged to toxin A+B+ strain, and all ST37 belonged to toxin A-B+ strain. ST39 (7/10) and ST26 (6/9) strains were mostly non-toxigenic. ST274 was a novel ST type, but negative for toxin detection. Strain information was submitted to the *C. difficile* MLST databases at the Oxford University, United Kingdom (Li, 2014). ST201 was toxin A+B+ strain and also positive for binary toxin, and the *tcdC* gene sequence revealed an 18-base pair deletion (nucleotides 330–347) located in *tcdC* (682bp, GenBank accession no. KM609431.1).

### Temporal Distribution and Outbreak of HOHA-CDI

The distribution of HOHA-CDI cases by month and week as well as by ICU are presented in **Figure 2**. Patient enrollment started on June 1, 2013 and the first confirmed CDI case was reported in mid-June 2013. A 15-case cluster of HOHA-CDI with different ST types (ST54, *n* = 7; ST26, *n* = 2; ST39, *n* = 2; ST3, *n* = 1; ST33, *n* = 1; ST133, *n* = 1; ST201, *n* = 1) was detected in February 2014. Of these cases, 8 occurred in the NICU, 5 in the GICU and 2 in the NSICU. Of note, among all ST54 strains (*n* = 14) identified in this study, 50% (*n* = 7) were linked to a CDI outbreak occurring in the NICU (*n* = 5) and GICU (*n* = 2) in February 2014 (**Figure 2**).

## Discussion

This study generated important information on the incidence rate and strains of HOHA-CDI among ICU patients at Xiangya Hospital in Changsha, China. During the 15-month study period, the incidence rate of HOHA-CDI among adult ICU patients was 14.1 cases per 10,000 patients-days. The rate was highest in the Neurology ICU. No cases were identified from the Respiratory ICU. *C. difficile* ST54 was the most common MLST type associated with strains circulating in the hospital, but was not associated with poorer patient outcomes. The detected case-cluster suggests possible gaps in infection control in the ICUs.

The overall incidence rate observed during our 15-month study was lower than estimated rates from another study which was 25.2 cases per 10,000 ICU days in China (Wang et al., 2014), but higher than the 2010 hospital-wide CDI incidence rate estimated from the National Hospital Surveillance Network (NHSN) in the U.S. of 7.4 per 10,000 patient-days (McDonald et al., 2012). Findings from a research project in 37 acute care hospitals in 14 European countries showed a hospital-wide rate of 3.7 (0.6–18.5) per 10,000 patient-days (van Dorp et al., 2016), and estimates from two hospital-wide projects in Perth, Australia showed rates of 3.9–16.3 per 10,000 patient-days in 2011 and 2012 (Foster et al., 2014). Global variability in incidence rates could be due to differences in surveillance sensitivity, testing methods, and patient-related factors as well as variability in infection control practices and distribution of toxigenic CDI strains. The finding of zero cases in the Respiratory ICU in our study could be related to one or more of these factors, including differences in adherence to surveillance protocols as well as in patient characteristics.

As previously mentioned, patients with diarrhea are not routinely tested for *C. difficile* in China (Li et al., 2014), and most CDI case-patients are identified by subjective judgment and clinical assessment of patient characteristics. Consequently, outside of a few research studies, the true burden of HOHA-CDI in China is very difficult to estimate. Required routine testing and implementation of HOHA-CDI reporting policies could help address this limitation (Murphy et al., 2012). With a high incidence, CDI detection is probably much more important in terms of monitoring effective intervention strategies as well as ensuring appropriate case management. Additionally, data on colectomies for toxic megacolon as well as sepsis cases and deaths associated with colonic pseudomembranes were not available. The lack of a systematic approach to identify cases could result in adverse patient outcomes. Future investigations aimed at capturing information about CDI diagnostic practices in China, including the proportion of cases diagnosed empirically or by laboratory testing, would be informative. Findings from such investigations could encourage the development and implementation of standard diagnostic criteria.

Based on the MLST analysis, 17 different STs were recognized in the study population; ST54 was the most common type. This distribution is similar to previous studies in China which identified the following strains: ST54 (23%), ST35 (19.3%), ST37 (9.9%) (Chen et al., 2014). Our previous study on CDI among patients with hospital-acquired pneumonia showed that the predominant types of *C. difficile* were ST54 (20%), ST37 (15.6%), and ST3 (9.4%) (Li et al., 2017). Although the North American/European epidemic strain RT027/ST1 was not detected, ST201, a toxin A+B+ strain that also contains a gene for binary toxin and has been associated with severe diarrhea (Li et al., 2015), was detected. This latter strain was similar to ST11 (RT 078), a type associated with community-acquired CDI commonly found in Europe (Knetsch et al., 2014).

We were unable to identify the cause of the outbreak of ST54 in the GICU and NICU in February 2014. These seven confirmed cases met our HOHA-CDI outbreak definition. However, given the temporal distribution of cases, separate introductions of the same strain could have occurred in the two ICUs. Of note, all patients linked to the outbreak were promptly treated by Vancomycin or Metronidazole. Methods to control the spread of *C. difficile* in hospitals include improving hand hygiene compliance, reducing unnecessary antibiotic prescribing, training healthcare employees to correctly care for active infections, and training hospital environmental services employees how to remove *C. difficile* from hospital environments.

## Limitations

Although there can be some under-detection of culture relative to nucleic acid amplification tests, the prospective enrollment of suspect CDI-diarrhea is a strength of this study. At the same time, a longer study will likely be needed in order to fully understand the variability of CDI detection across the four ICUs. Monitoring adherence to surveillance protocols as well as infection control measures will be helpful in describing differences in ICU CDI incidence rates. Also, we were unable to capture specific information on the dates and duration of antibiotic usage, particularly prior to hospitalization. Active monitoring and recording antibiotic use before and after patients’ hospital and ICU admission will be critical for future analyses. Finally, since the project was implemented in four ICUs at a single teaching hospital, the results may not represent HOHA-CDI incidence in other hospitals in China.

## Conclusion

HOHA-CDI occurs among ICU patients at this teaching hospital, supporting the importance of routine testing for CDI. Information on strain distribution can help detect CDI outbreaks. Detection of ST54 strain in a temporal cluster suggests possible gaps in infection control practices that should be investigated and addressed as needed. We recommend prospective monitoring and laboratory-based CDI surveillance in hospital ICUs.

## Author Contributions

CL, AW, YL, YH, CR, and JK conceived the experiments. CL, SL, XM, JD, and AW conducted the experiments. CL, SL, and JR analyzed the data. CL, YL, YH, JR, and CR drafted manuscript. CL, JR, and AW finalized the manuscript. All authors reviewed and approved the final manuscript.

## Disclaimer

The findings and conclusions in this report are those of the authors and do not necessarily represent the official position of US CDC.

## Conflict of Interest Statement

The authors declare that the research was conducted in the absence of any commercial or financial relationships that could be construed as a potential conflict of interest.
